# Stress, Burnout and Study-Related Behavior in University Students: A Cross-Sectional Cohort Analysis Before, During, and After the COVID-19 Pandemic

**DOI:** 10.3390/brainsci15070718

**Published:** 2025-07-04

**Authors:** Verena Dresen, Siegmund Staggl, Laura Fischer-Jbali, Markus Canazei, Elisabeth Weiss

**Affiliations:** Department of Psychology, University of Innsbruck, 6020 Innsbruck, Austria; verena.dresen@uibk.ac.at (V.D.); siegmund.staggl@uibk.ac.at (S.S.); laura.fischer-jbali@uibk.ac.at (L.F.-J.); markus.canazei@uibk.ac.at (M.C.)

**Keywords:** stress, burnout, study-related behavior, COVID-19, students

## Abstract

**Background/Objectives:** The COVID-19 pandemic intensified stress among students, though its impact on burnout symptoms remains mixed. Previous research emphasized examining both study-related behavior such as academic engagement and burnout for a fuller understanding of students’ well-being in the wake of the COVID-19 pandemic. **Methods:** In this cross-sectional study we examined stress, burnout, study-related behavior, and typical coping patterns among three cohorts of university students before (2016), at the start of (2020), and after (2024) the pandemic, with 1016 students participating. **Results:** Perceived stress was significantly higher during the pandemic but returned to pre-COVID-19 levels afterward. Depression scores remained stable across cohorts. Burnout symptoms, particularly cynicism and academic efficacy, were significantly lower in the COVID-19 cohort. Study commitment, including subjective importance of studying, academic goals/ambition, willingness to exert oneself, and striving for perfection were lower during and after the pandemic than before. Emotional distancing peaked in 2020, suggesting disengagement as a coping strategy. Pre-COVID-19 students exhibited higher active coping scores than the COVID-19 and post-COVID-19 cohorts, while satisfaction with studies was highest post-pandemic, likely due to the return of in-person academic and social experiences. **Conclusions:** These findings reveal fluctuations in students’ stress, burnout, and study-related behavior over time. While stress-levels have normalized, study commitment and typical coping patterns such as active coping remain altered, indicating the pandemic’s lasting impact on students’ academic behavior and mental health.

## 1. Introduction

Previous research has established that stress during academic training constitutes a significant issue for many students [1,2]. Persistent stress is associated with diminished academic performance and increased dropout rates [3] and represents a major risk factor for students’ mental and physical health [4]. Several meta-analyses have demonstrated that the experience of stress among students intensified during the COVID-19 pandemic, with up to two-thirds of students reporting high levels of stress [5,6]. Recent findings suggest that, in addition to concerns regarding infection risk, the transition from in-person to remote learning, the increased flow of information, uncertainty regarding examination modalities, the blurring of boundaries between academic and personal life, financial insecurity, concerns about academic progress, and particularly social isolation—often accompanied by feelings of loneliness—adversely affected students’ well-being (e.g., [7,8]).

Chronic stress is also a significant risk factor for the development of burnout [9]. To date, no universally accepted definition of burnout exists, and the syndrome is not classified as a distinct psychiatric disorder in clinical diagnostic systems such as the International Classification of Diseases and Related Health Problems (ICD-11 [10]) or the Diagnostic and Statistical Manual of Mental Disorders (DSM-5 [11]). The most widely recognized definition by Maslach and Jackson [12] conceptualizes burnout as comprising three core dimensions: emotional exhaustion, depersonalization, and reduced personal accomplishment [9]. In the academic context, burnout manifests primarily as a sense of exhaustion due to perceived excessive academic demands. Additionally, affected individuals often develop a detached and cynical attitude toward their studies and experience self-doubt regarding their abilities and productivity, contributing to a perceived sense of incompetence [13].

Even prior to the COVID-19 pandemic, research indicated that a substantial proportion of university students experienced academic burnout (meta-analysis [14]). Prevalence rates varied across disciplines, with rates ranging from 16% to 27% among students of law, teacher education, business, social sciences, arts, and psychology [15,16], and reaching 45–56% among medical students (meta-analysis [17,18]).

A recent meta-analysis by Abraham et al. [19], which synthesized data from 31 countries and 26.500 students, found that the average prevalence rates for emotional exhaustion, cynicism, and reduced academic efficacy were 56.3%, 55.3%, and 41.8%, respectively. Students in low-income countries exhibited the highest prevalence rates, exceeding 80%. Notably, significant differences were observed across academic disciplines, with medical students consistently reporting the highest burnout levels.

Longitudinal and cohort studies comparing pre-pandemic and pandemic periods largely identified a significant increase in burnout symptoms attributable to the COVID-19 pandemic [20,21,22,23]. However, some studies reported a decline in burnout symptoms [24], while others found no substantial changes [25]. Certain field-specific factors, such as the cancelation of medical internships, may have influenced these findings [26].

There remains a paucity of cohort and longitudinal studies examining stress and burnout among students in the post-pandemic period and the subsequent transition back to in-person learning. The existing evidence is heterogeneous. Zhu et al. [27] reported that post-pandemic burnout prevalence may not only have improved compared to pandemic levels but may have even fallen below pre-pandemic levels from 2018. Conversely, other studies have indicated increased burnout rates and lower academic engagement relative to pre-pandemic levels [20,23]. To gain a more comprehensive understanding of students’ academic well-being, several scholars advocate for the simultaneous examination of academic engagement and burnout [28,29].

Beyond academic engagement, additional behavioral and experiential patterns may contribute to and sustain burnout. Based on a salutogenic framework, Schaarschmidt et al. [16] identified a healthy behavioral pattern, an unambitious pattern, and two risk patterns associated with overexertion and burnout. Voltmer et al. [30] demonstrated in a longitudinal study that during the COVID-19 pandemic, the proportion of students exhibiting a healthy behavioral and experiential pattern declined, while an increasing number of students exhibited a risk pattern indicative of overexertion.

Against this background, the present study examined stress and burnout symptoms among university students before, during, and after the pandemic. Additionally, we analyze differences in study-related behavior across the three cohorts using a modified Version of the Assessment of Work-related Behavior and Experience Patterns (AVEM [16]) adapted for students. The modified AVEM assesses individual experiences of study-related stress and typical coping behavior across three key domains: study commitment, stress resilience, and emotional well-being.

## 2. Materials and Methods

### 2.1. Study Procedure and Participants

The study sample consisted of three cohorts of German-speaking students from various Austrian and German universities. Data collection was conducted during the examination periods in June and July. The pre-COVID-19 cohort was surveyed in 2016, the COVID-19 cohort during the COVID-19 pandemic in 2020, and the post-COVID-19 cohort in 2024. Ethical approval was obtained from the Ethics Review Boards of the University of Innsbruck and the University of Graz. Informed consent was obtained from all individual participants included in the study.

A total of 1126 students participated in the online survey. Recruitment was carried out via social media, university courses, and the University of Innsbruck’s mailing list. The inclusion criterion was active enrollment in a university program. Participants with incomplete questionnaire responses were excluded (*n* = 110), resulting in a final sample of 1016 students (pre-COVID-19 cohort: *n* = 364; COVID-19 cohort: *n* = 350; post-COVID-19 cohort: *n* = 302). The participants’ ages ranged from 18 to 49 years (*M* = 23.82, *SD* = 3.77).

### 2.2. Measures

#### 2.2.1. Center for Epidemiologic Studies Short Depression Scale (CES-D-R15)

The CES-D-R15 [31] assesses depressive symptoms experienced in the past week using 15 items (e.g., “In the past week, I felt sad”). The scale demonstrates high internal consistency across different samples (α = 0.88 to 0.95) and is rated on a four-point Likert scale ranging from 0 (rarely or never, less than 1 day) to 3 (most of the time, 5 to 7 days). A higher score indicates greater depressive symptoms. The internal consistency of the CES_D-R in the current study was high (Cronbach’s α = 0.87).

#### 2.2.2. Perceived Stress Scale (PSS-10)

Perceived stress was measured using the German version of the Perceived Stress Scale (PSS-10; [4]), which evaluates the extent to which individuals perceived their lives as unpredictable, uncontrollable, and overwhelming over the past month. The scale captures chronic stress resulting from various life circumstances, including academic stressors. It consists of 10 items (e.g., “In the last month, how often have you felt nervous and stressed?”), rated on a five-point frequency scale from 0 (never) to 4 (very often). Higher scores indicate higher levels of perceived stress. In the present study, the PSS-10 demonstrated good internal consistency reliability, with Cronbach’s alpha = 0.92.

#### 2.2.3. Maslach Burnout Inventory—Student Survey German Version (MBI-SS)

Burnout symptoms were assessed using the German version of the Maslach Burnout Inventory—Student Survey (MBI-SS; [32]). The questionnaire consists of 15 items that are grouped into three subscales. The *Emotional Exhaustion subscale* consists of 5 negatively worded items (e.g., “I feel emotionally drained by my studies.”). The *Cynicism/Depersonalization subscale* consists of 4 negatively items (e.g., “I doubt the significance of my studies.”) and the *Academic Efficacy subscale* consists of 6 positively worded items (e.g., “I can effectively solve problems that arise in my studies.”). All items were rated on a seven-point frequency scale ranging from 0 (never) to 6 (daily). High emotional exhaustion and cynicism scores, combined with low academic efficacy (reverse-scored), suggest burnout. In the present study, the internal consistency of the MBI-SS scales was high (Cronbach’s α = 0.87 for *Emotional Exhaustion* subscale; α = 0.71 for *Cynicism/Depersonalization* subscale; α = 0.79 for the *Academic Efficacy* subscale.

#### 2.2.4. Modified Version of the Assessment of Work-Related Behavior and Experience Patterns (AVEM) Adapted for Students

In the current study we used a modified version of the AVEM adapted for students to address the individual experiences of study-related stress and typical coping behaviors. We made slight modifications to the original AVEM instructions, for example replacing the word “work” with “study” or replacing “professional development” with “previous development at school and university”. The questionnaire consists of 66 items and assesses an individual’s study-related behavior across the three key domains: *study commitment*, *stress resilience*, and *emotional well-being* [16]. These domains are measured using 11 subscales, each comprising six items rated on a 5-point Likert scale from 1 (“strongly disagree”) to 5 (“strongly agree”), with a maximum subscale score of 30 points. Higher scores indicate a stronger manifestation of the respective characteristic. In the current paper Cronbach’s alpha for the 11 subscales showed high internal consistency, ranging between 0.77 and 0.88. The 11 subscales are assigned to the three key domains as follows:

Study Commitment (Subscales 1–5): This dimension reflects the extent to which individuals are committed to their study, including subjective importance of studying, academic goals/ambition, willingness to exert oneself, striving for perfection, and emotional distancing (e.g., “Studying is the most important thing in my life.”).

Stress Resilience (Subscales 6–8): This factor represents an individual’s ability to cope with study-related stress, encompassing emotional distancing (dual loading), tendencies towards resignation, proactive problem-solving, and inner balance (e.g., “Nothing upsets me easily.”).

Emotional Well-Being (Subscales 9–11): This domain evaluates a person’s affective response to study-related challenges, including satisfaction with studies, overall life satisfaction, and perceived social support (e.g., “So far, my studies have been quite successful.”).

### 2.3. Statistics

Descriptive data are presented as means (*M*) and standard deviations (*SD*). Pearson’s chi-square tests or Fisher’s exact tests with Monte Carlo estimation were used to compare the demographic characteristics (gender, level of education, university courses) of the three cohorts (pre-COVID-19 [first cohort], COVID-19 [second cohort], post-COVID-19 [third cohort]). A univariate analysis of variance (ANOVA) was used to examine age differences between the three cohorts of students. As we observed, age differences between the students in the three cohorts, differences between the three groups for CES-D-R15, PSS-10, MBI, and the three domains of the AVEM were compared by means of univariate/multivariate analysis of covariance ((M)ANCOVAs). Post hoc pairwise comparisons with Bonferroni corrections were conducted for significant effects in the (M)ANCOVAs, and effect sizes are reported as partial eta-squared (η_p_^2^). For the statistical significance test, the alpha level was set a priori at 0.05. All statistical procedures were performed in SPSS 26.

## 3. Results

### 3.1. Sample Characteristics

The age range of the sample was between 18 and 49 years (mean age: *M* = 23.82 ± 3.77). A total of 733 female, 280 male, and 3 gender diverse students participated in the study. Of the participants, 71.8% (*n* = 692) were undergraduate students, while 26.9% (*n* = 259) were enrolled in a Master’s program and 1.3% (*n* = 13) students in a PhD program. Most students (41.3%) were studying psychology (*n* = 419) or social sciences and humanities (29.9%). Table 1 presents the sociodemographic and study-related information.

There was a significant difference in the age of the students between the three cohorts (*F*(2, 1013) = 20.829, *p* < 0.001, η_p_^2^ = 0.039). Significant differences could also be found for gender (Fisher’s exact tests with Monte Carlo estimation: *p* = 0.009), additional job (χ^2^(2, *n* = 1016) = 38.585, *p* < 0.001), the level of education (χ^2^(4, *n* = 964) = 51.382, *p* < 0.001), and the university courses (χ^2^(10, *n* = 1016) = 131.067, *p* < 0.001). No significant difference was found for marital status (χ^2^(2, *n* = 1016) = 1.512, *p* < 0.470), and children (Fisher’s exact tests with Monte Carlo estimation: *p* = 0.519).

Table 2 shows the descriptive statistics for the clinical scales and study-related scales for the three cohorts.

### 3.2. Stress and Depression

A univariate analysis of covariance (ANCOVA) using age as covariate showed a significant difference between the three cohorts on the PSS-10 (*F*(2, 1012) = 4.657, *p* = 0.010, η_p_^2^ = 0.009). Bonferroni-corrected post hoc tests revealed that the COVID-19 cohort exhibited a statistically significantly higher PSS-10 stress-score compared to the pre-COVID-19 cohort (*p* < 0.001). No significant differences were observed for the other pairwise comparisons (all values of *p* > 0.1).

Regarding depression scores (CES-D-R15), no significant differences were found among the three cohorts (*F*(2, 1012) = 1.181, *p* = 0.307, η_p_^2^ = 0.002).

**Figure 1:**
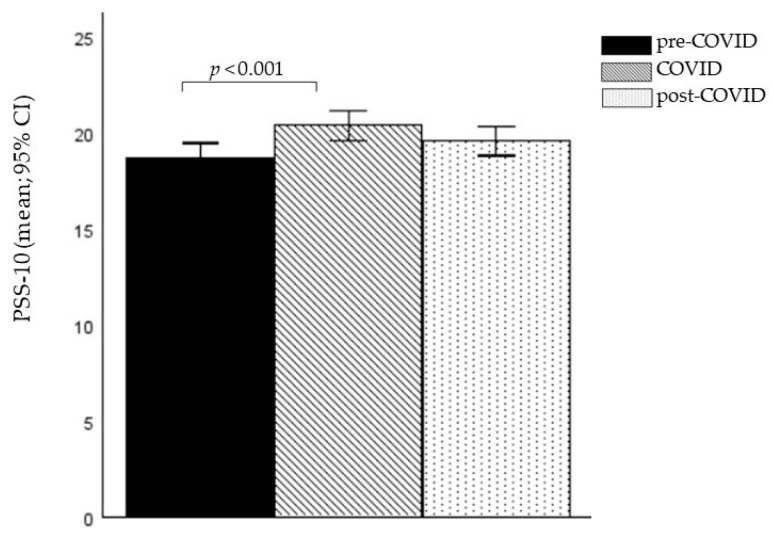
Group differences in the PSS-10 across the three cohorts. Note: the figure shows means and 95% confidence intervals of the means (95% CI); PSS-10 = Perceived Stress Scale.

The means and 95% confidence intervals of the means (95% CI) of the PSS-10 for the three cohorts are presented in Figure 1.

### 3.3. Burn-Out Symptoms

A multivariate analysis of covariance (MANCOVA) based on the three MBI scales with the three cohorts as the between-subjects factor and age as the covariate revealed a significant multivariate main effect group (*F*(6, 2022) = 11.867, *p* < 0.001, η_p_^2^ = 0.034). Subsequent univariate analyses indicated significant effects for the two scales *Cynicism/Depersonalization* (*F*(2, 1012) = 16.184, *p* < 0.001, η_p_^2^ = 0.031) and *Academic Efficacy* (*F*(2, 1012) = 4.887, *p* = 0.008, η_p_^2^ = 0.010) and a trend towards significance for the scale *Emotional Exhaustion* (*F*(2, 1012) = 2.873, *p* = 0.057, η_p_^2^ = 0.006).

Bonferroni-corrected post hoc test revealed significantly lower *Cynicism/Depersonalization* scores in the COVID-19 cohort compared to both the pre-COVID-19 cohort (*p* < 0.001) and post-COVID-19 cohort (*p* = 0.001). No difference was found between the pre-COVID-19 and post-COVID-19 cohort (*p* = 0.282).

In the *Academic Efficacy* scale, the pre-COVID-19 cohort showed significantly higher scores than the COVID-19 cohort (*p* = 0.009) and a trend towards higher scores than the post-COVID-19 cohort (*p* = 0.082). No significant difference was found between COVID-19 and post-COVID-19 cohort (*p* > 1).

**Figure 2:**
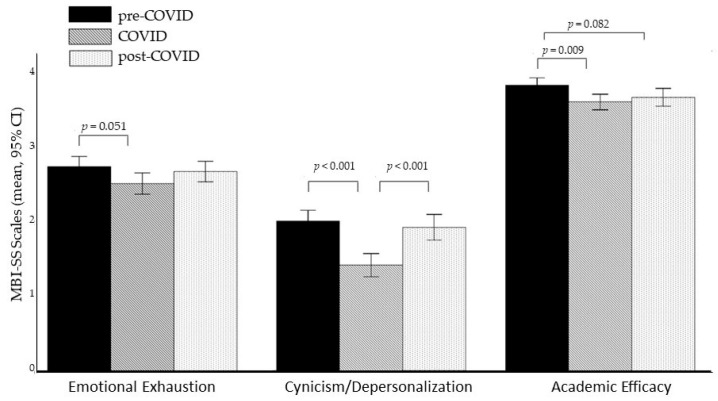
Group differences in the MBI-SS scales. Note: Figure shows means and 95% confidence intervals of the means (95% CI); MBI-SS = Maslach Burnout Inventory—Student Survey.

Only a trend towards higher *Emotional Exhaustion* scores in the pre-COVID-19 cohort compared to the COVID-19 cohort was observed (*p* = 0.051), while no other pairwise comparisons were significant (all values of *p* > 0.1, see Table 2 and Figure 2).

### 3.4. Assessment of Study Behavior and Experience Patterns

Three separate MANCOVAs were performed for the three AVEM domains.

#### 3.4.1. Study Commitment Domain (AVEM)

A multivariate analysis of covariance (MANCOVA) was conducted on the five subscales within the *Study Commitment* domain, with cohort as the between-subjects factor and age as the covariate. A significant multivariate main effect of cohort was observed (*F*(10, 2018) = 11.297, *p* < 0.001, η_p_^2^ = 0.053). Univariate analyses revealed significant effects for all five subscales: *Perceived Significance of Study* (*F*(2, 1012) = 5.667, *p* = 0.004, η_p_^2^ = 0.011), *Academic Goals/Ambition* (*F*(2, 1012) = 19.425, *p* < 0.001, η_p_^2^ = 0.037), *Commitment* (F(2, 1012) = 22.177, *p* < 0.001, η_p_^2^ = 0.042), *Striving for Perfection* (F(2, 1012) = 35.967, *p* < 0.001, η_p_^2^ = 0.066), and *Emotional Distancing* (*F*(2, 1012) = 5.247, *p* = 0.005, η_p_^2^ = 0.010).

Bonferroni-corrected post hoc tests indicated that the COVID-19 cohort had significantly lower scores on *Academic Goals/Ambition*, *Commitment*, and *Striving for Perfection* (all values of *p* < 0.001), but higher scores on the subscale *Emotional Distancing* (*p* = 0.004) compared to the pre-COVID-19 cohort. No significant difference was found for the *Perceived Significance of Study* subscale between the pre-COVID-19 and COVID-19 cohort (*p* > 0.1).

Compared to the post-COVID-19 cohort, the COVID-19 cohort exhibited significantly higher scores in the subscale *Perceived Significance of Study* (*p* = 0.017) but lower *Striving for Perfection* scores (*p* = 0.006). Post hoc tests for the other three subscales (Academic Goals/Ambition, Commitment, and Emotional Distancing) did not reach significance between the Post-COVID-19 and COVID-19 cohorts (all *p*’s > 0.1).

Compared to the post-COVID-19 cohort the pre-COVID-19 cohort demonstrated significantly higher scores in the subscales *Perceived Significance of Study* (*p* = 0.005), *Academic Goals/Ambition*, *Commitment*, and *Striving for Perfection* (all *p*’s < 0.001), with no significant difference in the subscale *Emotional Distancing*(all *p*’s > 0.1).

**Figure 3:**
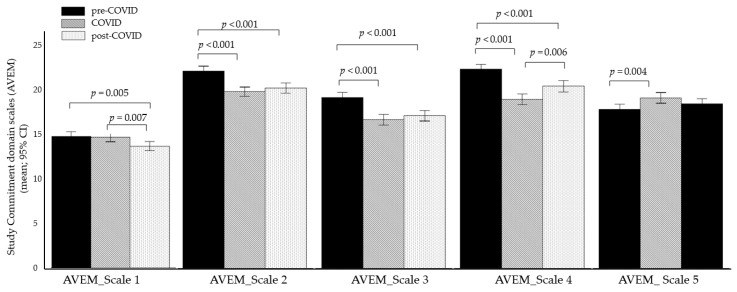
Group differences in the *Study Commitment* domain scales (AVEM). Note: the figure shows means and 95% confidence intervals of the means (95% CI); modified AVEM = Assessment of Study-related Behavior and Experience Pattern; AVEM_Scale1 = *Perceived Significance of Study*; AVEM_Scale 2 = *Academic Goals/Ambition*; AVEM_Scale 3 = *Commitment*; AVEM_Scale 4 = *Striving for Perfection*; AVEM_Scale 5 = *Emotional Distancing*.

The means and 95% confidence intervals of the means (95% CI) of the *Study Commitment* domain scales for the three cohorts are presented in Figure 3.

#### 3.4.2. ‘Resistance to Stress’ Domain (AVEM)

A MANCOVA on the three scales of the *Resistance to Stress* subscales with cohort as the between-subjects factor and age as the covariate revealed a significant multivariate main effect of cohort (*F*(6, 2022) = 5.913, *p* < 0.001, η_p_^2^ = 0.017). Univariate analyses indicated a significant effect for *Active Coping* (*F*(2, 1012) = 13.823, *p* < 0.001, η_p_^2^ = 0.027), but no significant differences in the *Tendency to Resignation* (*F*(2, 1012) = 1.006, *p* = 0.366, η_p_^2^ = 0.002) or *Balance and Emotional Stability* (*F*(2, 1012) = 0.060, *p* = 0.942, η_p_^2^ < 0.001).

Bonferroni-corrected post hoc tests for *Active Coping* showed that the pre-COVID-19 cohort had significantly higher scores than both the COVID-19 (*p* < 0.001) and post-COVID-19 cohorts (*p* < 0.001) with no significant difference between the COVID-19 and post-COVID-19 cohorts (*p* = 1).

**Figure 4:**
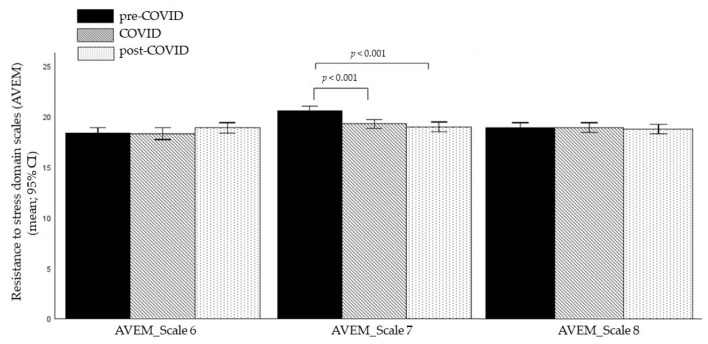
Group differences in the *Resistance to Stress* domain scales (AVEM). Note: the figure shows means and 95% confidence intervals of the means (95% CI); modified AVEM = Assessment of Study-related Behavior and Experience Pattern; AVEM_Scale 6 = *Tendency to Resignation*; AVEM_Scale 7 = *Active Coping*; AVEM_Scale 8 = *Balance and Emotional Stability*.

The means and 95% confidence intervals of the means (95% CI) of the three *Resistance to Stress* scales (AVEM) for the three cohorts are presented in Figure 4.

#### 3.4.3. ‘Emotional or Subjective Well-Being’ Domain (AVEM)

A MANCOVA on the three *Emotional or Subjective Well-Being* subscales (AVEM), using cohort as the between-subjects factor and age as the covariate, revealed a significant multivariate main effect of cohort (*F*(6, 2022) = 5.305, *p* < 0.001, η_p_^2^ = 0.015). Subsequent univariate analyses indicated a significant effect of the scale *Satisfaction with Studies* (*F* (2, 1012) = 13.418, *p* < 0.001, η_p_^2^ = 0.016) but no significance for the scales *Life Satisfaction* (*F*(2, 1012) = 0.129, *p* = 0.879, η_p_^2^ = 0.000) and *Perceived Social Support* (*F*(2, 1012) = 0.020, *p* = 0.980, η_p_^2^ = 0.000).

Bonferroni-corrected post hoc tests for the significant *Satisfaction with Studies* scale revealed that the pre-COVID-19 cohort had significantly lower scores than both the COVID-19 (*p* = 0.033) and post-COVID-19 cohorts (*p* <.001). Additionally, the COVID-19 cohort exhibited significantly lower scores than the post-COVID-19 cohort (*p* = 0.017).

**Figure 5:**
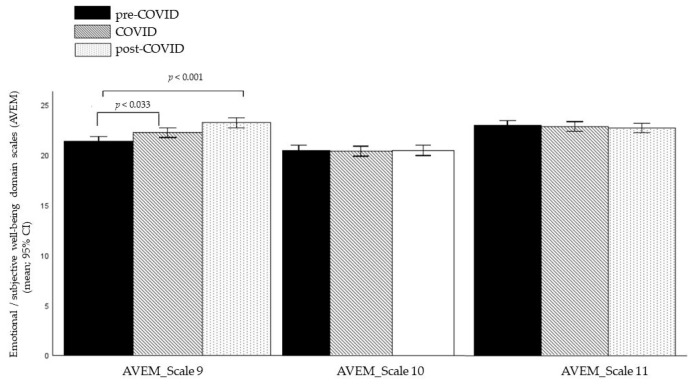
Group differences in the *Emotional or Subjective Well-Being* domain scales (AVEM). Note: the figure shows means and 95% confidence intervals of the means (95% CI); modified AVEM = Assessment of Study-related Behavior and Experience Pattern; AVEM_Scale 9 = *Satisfaction with Studies*; AVEM_Scale 10 = *Life Satisfaction*; AVEM_Scale 11 = *Perceived Social Support*.

The means and 95% confidence intervals of the means (95% CI) of the three *Emotional or Subjective Well-Being* scales (AVEM) for the three cohorts are presented in Figure 5.

## 4. Discussion

The present cross-sectional study examined perceived symptoms of stress, depression, burnout, and study-related behavior among university students before, during, and after the COVID-19 pandemic: pre-COVID-19 cohort in 2016, COVID-19 cohort in 2020, and post-COVID-19 cohort in 2024. The findings contribute to the growing body of literature examining the long-term impact of the pandemic on students’ psychological well-being and study-related attitudes over time.

### 4.1. Symptoms of Stress and Depression

Consistent with prior research [5,6], the results indicate that perceived stress levels were significantly higher during the COVID-19 pandemic compared to the pre-COVID-19 period, although the effect size of this difference was small. This aligns with the documented challenges students faced, such as remote learning, social isolation, and increased academic uncertainty [33]. However, post-COVID-19 stress levels did not significantly differ from the pre-pandemic period, suggesting a potential return to baseline stress levels as students transitioned back to in-person learning.

Interestingly, no significant differences in depression scores were found across the three cohorts, which contrasts with the results from a recently published meta-analyses indicating heightened depressive symptoms during the pandemic [19]. This finding could suggest that while students experienced heightened stress during COVID-19, their depressive symptoms may have been more stable over time, possibly due to individual coping mechanisms, social support, or adaptation to the crisis. The current study consisted mainly of students studying psychology or social sciences and humanities. Students in these academic disciplines may have more knowledge about mental health and stress coping techniques than the average student population. However, the literature about the mental health of students from different university courses is very inconsistent, and several factors may account for these inconsistent results, such as the female ratio in the study population as well as other heterogeneity of the study populations such as demographic factors (e.g., age, socioeconomic status, geographical location, or cultural influence) and a wide variety of course structures (e.g., [34,35,36]). Another potential explanation is the use of a short general depression measure that may not fully capture the nuanced emotional states associated with pandemic-related distress.

### 4.2. Burnout Symptoms

Burnout, particularly cynicism and reduced academic efficacy, varied significantly across the three cohorts, but again the effect sizes of these differences were small. Students in the COVID-19 cohort reported significantly lower cynicism/depersonalization compared to both the pre-COVID-19 and post-COVID-19 cohorts. This unexpected finding might be attributed to the altered academic environment during the pandemic, where online learning and increased flexibility may have temporarily alleviated certain academic pressures, leading to reduced cynicism towards studies. In addition, in the COVID-19 cohort, less students were able to pursue a part-time job due to the restrictions during the COVID-19 pandemic. Conversely, cynicism rebounded post-pandemic, approaching pre-COVID-19 levels, potentially reflecting lingering frustrations with the transition back to conventional academic structures. Furthermore, in the post-COVID-19 group, students were significantly more likely to have a part-time job in addition to their studies

For academic efficacy, the pre-COVID-19 cohort reported significantly higher scores than the COVID-19 cohort and with a trend towards higher scores compared to the post-COVID-19 cohort. This suggests that students during the pandemic may have had a lower sense of accomplishment, potentially due to problems in adapting to an unprecedented academic situation. However, the lack of significant differences between the COVID-19 and post-COVID-19 cohorts indicates that this lower sense of accomplishment may have persisted beyond the immediate crisis. Nevertheless, one has to keep in mind that the pre-COVID-19 cohort had a higher percentage of medical students compared to all other cohorts. Research indicates that the demanding nature of medical education, characterized by extensive workloads, high expectations, and continuous exposure to clinical environments, may have contributed to higher scores in the scale academic efficacy among medical students compared to their peers in other fields [17,36].

Although emotional exhaustion showed only a trend towards significance, the slight decrease in exhaustion scores in the COVID-19 cohort compared to the pre-COVID-19 cohort is in contrast to previous findings that pandemic-related stressors contributed to higher fatigue and emotional strain among students (e.g., [23]). This could be due to the fact that students were not able to pursue additional part-time jobs or extensive leisure activities during the COVID-19 pandemic and were therefore less emotionally exhausted. Overall, the findings regarding the impact of the pandemic on burnout symptoms are heterogeneous. While some studies report significant increases in burnout levels [20,21,22,23], others show only marginal changes or even declines [24,25]. The causes assumed by the authors are often chosen in a context-dependent manner and highlight different influences of the COVID-19 pandemic on the study situation (e.g., gender, first-year students vs. more experienced students, field of study such as medicine vs. other disciplines, cancelation of internships). On the one hand, social restrictions, planning uncertainties, and technical challenges can contribute to the development of burnout, while on the other hand, flexible learning formats, the elimination of commuting times, and the absence of clinical internships or additional part-time jobs may have a relieving effect and reduce the risk of burnout.

### 4.3. Study-Related Behavior and Experience

The findings regarding students’ commitment to study revealed important shifts in study-related attitudes, although the observed changes were associated with mainly small effect sizes. Study commitment scores, including academic goals/ambition and striving for perfection, were notably lower during and after the pandemic compared to pre-COVID-19 levels, suggesting that the pandemic may have diminished students’ motivation and engagement with their academic goals. This finding is in line with previous studies indicating reduced academic motivation following prolonged periods of remote learning [23]. Additionally, emotional distancing was highest in the COVID-19 cohort, reflecting students’ potential disengagement as a coping mechanism.

In terms of stress resistance, the pre-COVID-19 cohort demonstrated significantly higher active coping scores than both the COVID-19 and post-COVID-19 cohorts, suggesting a decline in students’ ability to actively manage stress related to the pandemic. The lack of significant differences between the COVID-19 and post-COVID-19 cohorts implies that the decline in active coping strategies may be a lasting consequence of the pandemic rather than a temporary disruption. In contrast, no significant differences were found for resignation tendencies or emotional stability, indicating that while students’ proactive coping abilities declined, their overall emotional resilience remained relatively stable.

Satisfaction with studies was highest in the post-COVID-19 cohort. This might be attributed to a renewed sense of normality and the return of in-person academic and social experiences. However, life satisfaction and perceived social support remained stable across the three cohorts, implying that while academic satisfaction fluctuated, overall life perspectives remained relatively consistent. A longitudinal study before and after the onset of the pandemic examined the changes in students’ behavior and experience patterns using the three AVEM domains [30]. The proportion of students with a healthy pattern and a risk pattern for overexertion slightly declined, while those with an unambitious pattern and a risk pattern for burnout increased.

### 4.4. Limitations

One key limitation of this study is the unequal distribution of academic disciplines across the three cohorts, particularly the higher proportion of medical students in the pre-COVID-19 cohort. Given that medical students report significantly higher burnout rates than students from other disciplines [17,18], the pre-pandemic burnout scores may have been inflated due to the overrepresentation of this subgroup. This could have influenced the observed patterns, potentially underestimating the extent of burnout in the general student population.

Furthermore, the current study consisted mainly of psychology students and students majoring in social sciences and humanities. Previous studies could show that students in mental health-related majors were more aware of warning signs and resources for mental health issues compared to students in engineering, math and business majors [37,38]. This may limit the generalizability of our results, as the stress experience and coping mechanisms of students in these academic disciplines may not be representative of the general student population.

Another limitation is that the cross-sectional study design does not allow for causal inferences. While cohort comparisons provide valuable insights, longitudinal data tracking of individual students across time would offer a more precise understanding of how stress and burnout trajectories evolve.

Lastly, the gender ratio was not balanced across the whole sample, as the majority of students (76.7%) were female. The body of existing literature consistently underscores the presence of a gender gap in mental health, with evidence suggesting that women and men differ in their susceptibility to stress [39] and their physiological and psychological responses to stress [40]. Moreover, women are reported to experience a 2- to 3-fold higher prevalence of depression and anxiety disorders compared to men [41]. However, research on gender differences in mental health within student populations presents more heterogeneous findings. Several studies have reported higher levels of stress, depression, or anxiety among female students [42,43,44]. In contrast, other studies have identified higher rates of depression among male students [45,46,47], while some have found no significant gender differences [48]. Research investigating whether the gender gap in mental health widened during the COVID-19 pandemic has generally reported no or only minor gender differences in stress, anxiety, depression, and overall mental health [49] (for a review, see [50]).

## 5. Conclusions and Implications

Overall, the study highlights significant fluctuations in students’ stress, burnout, and study-related behavior before, during, and after the pandemic. While perceived stress scores have returned to pre-pandemic levels, study commitment, including subjective importance of studying, academic goals/ambition, willingness to exert oneself, and striving for perfection, as well as typical coping patterns such as active coping, remain altered, suggesting that the effects of the COVID-19 pandemic on academic behavior and mental health still persist.

These results underscore the importance of long-term monitoring of student well-being and study-related behaviors, as well as the need for targeted interventions to support students in developing effective coping strategies and maintaining engagement in their academic goals. Universities should consider incorporating mental health support programs and fostering adaptive learning environments that balance academic demands with students’ well-being, particularly during periods of major transitions. Addressing students’ coping mechanisms and fostering resilience will be crucial in mitigating long-term academic and psychological consequences of the pandemic.

## Figures and Tables

**Table 1:** Sociodemographic and study-related information.

	Pre-COVID *n* = 364	COVID *n* = 350	Post-COVID *n* = 302	Total *n* = 1016
**Age** [years]: mean (*SD*)	23.17 (3.23)	23.53 (3.81)	24.95 (4.09)	23.82 (3.77)
**Gender (** ***n*** **,%)**				
female	249 (68.4%)	266 (76.0%)	218 (72.2%)	733 (72.2%)
Male	115 (31.6%)	84 (24.0%)	81 (26.8%)	280 (27.6%)
Diverse	0 (0%)	0 (0%)	3 (1.0%)	3 (0.3%)
**Marital status**				
Single/divorced	157 (43.1%)	167(47.7%)	137 (45.4%)	461(45.4%)
Married/with a life partner	207 (56.9%)	183 (52.3%)	165 (54.6%)	555 (54.6%)
**Children**				
Yes	6 (1.7%)	8 (2.3%)	9 (3%)	23 (2.3%)
No	358 (98.3%)	342 (97.7%)	293 97%)	993 (97.7%)
**Additional Job**				
Yes	181 (49.7%)	145 (41.4%)	198 (65.6%)	524 (51.6%)
No	183 (50.3%)	205 (58.6%)	104 (34.4%)	492 (48.4)
**Level of education (** ***n*** **,%)**				
Bachelor	247 (79.2%)	270 (77.1%)	175 (67.6%)	692 (71.8%)
Master	65 (20.8%)	71 (20.3%)	123 (27.6%)	259 (26.9%)
PhD	0 (0%)	9 (2.6%)	4 (1.8%)	13 (1.3%)
**University courses (** ***n*** **,%)**				
Psychology	85 (23.4%)	173 (49.4%)	161 (53.3%)	419 (41.3%)
Medicine	50 (13.7%)	26 (7.4%)	5 (1.7%)	81 (8.0%)
Law	40 (11.0%)	16 (4.6%)	5 (1.7%)	61 (6.0%)
Natural Science	37 (10.2%)	29 (8.3%)	8 (2.7%)	74 (7.3%)
Social Sciences and Humanities	110 (30.2%)	89 (25.4%)	105 (34.8%)	304 (29.9%)
Engineering and Technology	42 (11.5%)	17 (4.9%)	18 (6.0%)	77 (7.6%)

**Table 2:** Descriptive statistics for the clinical and study-related scales for the three cohorts.

	Pre-COVID Mean (*SD*)	COVID Mean (*SD*)	Post-COVID Mean (*SD*)
**CES-D-R15**	28.88 (9.37)	29.97(9.85)	29.36 (8.82)
**PSS-10**	18.7 (7.59)	20.38 (7.57)	19.57 (6.61)
**MBI-SS**			
*MBI_EE*	2.73 (1.31)	2.50 (1.35)	2.66 (1.21)
*MBI_Cyn/Dep*	1.99 (1.42)	1.41 (1.47)	1.91 (1.52)
*MBI_Effic*	3.81 (0.99)	3.59 (1)	3.66 (1.05)
**AVEM**			
***AVEM*** ***Study Commitment***			
Scale 1: *Perceived Significance*	14.57 (4.85)	14.45 (4.65)	13.48 (4.42)
Scale 2: *Academic Goals/Ambition*	21.37 (5.06)	19.13 (4.77)	19.52 (4.85)
Scale 3: *Commitment*	18.5 (5.36)	16.09 (5.45)	16.52 (4.95)
Scale 4: *Striving for Perfection*	21.57 (4.89)	18.31 (5.44)	19.72 (5.39)
Scale 5: *Emotional Distancing*	17.21 (5.37)	18.46 (5.51)	17.82 (4.88)
***AVEM Resistance to Stress***			
Scale 6: *Tendency to Resignation*	18.34 (5)	18.29 (5.5)	18.86 (4.51)
Scale 7: *Active Coping*	20.71 (4.43)	19.4 (4.09)	19.11 (4.23)
Scale 8: *Balance and Emotional Stability*	19 (5.01)	19.03 (4.62)	18.89 (4.06)
***AVEM Emotional or Subjective Well-Being***			
Scale 9: *Satisfaction with Studies*	21.32 (4.68)	22.19 (4.57)	23.19 (4.48)
Scale 10: *Life Satisfaction*	20.57 (5.09)	20.49 (4.81)	20.58 (4.56)
Scale 11: *Perceived Social Support*	23.29 (4.67)	23.19 (4.64)	23.04 (4.16)

Note: The table shows means (standard deviations). CES-D-R15 = Center for Epidemiologic Studies Short Depression Scale; PSS-10 = Perceived Stress Scale; MBI-SS = Maslach Burnout Inventory—Student Survey German Version; *MBI_EE* = Emotional Exhaustion subscale; *MBI_Cyn/Dep* = Cynicism/Depersonalization subscale; *MBI_Effic* = Academic Efficacy subscale; Modified AVEM = Assessment of Study-related Behavior and Experience Pattern.

## Data Availability

The datasets generated and/or analyzed during the current study are available from the corresponding author upon reasonable request. The data are not publicly available due to ethical restrictions.

## Abbreviations

The following abbreviations are used in this manuscript:

AVEM	Assessment of Work Behavior and Experience Patterns
CES-D-R15	Center for Epidemiologic Studies Short Depression Scale
MBI-SS	Maslach Burnout Inventory—Student Survey
MBI_EE	Emotional Exhaustion Subscale of the MBI-SS
MBI_Cyn/Dep	Cynicism/Depersonalization Subscale of the MBI-SS
MBI_Effic	Academic Efficacy of the MBI-SS
PSS-10	Perceived Stress Scale
